# The role of valence, dominance, and pitch in perceptions of artificial intelligence (AI) conversational agents’ voices

**DOI:** 10.1038/s41598-022-27124-8

**Published:** 2022-12-28

**Authors:** Victor Kenji M. Shiramizu, Anthony J. Lee, Daria Altenburg, David R. Feinberg, Benedict C. Jones

**Affiliations:** 1 School of Psychological Sciences & Health, University of Strathclyde, Glasgow, Scotland; 2 Division of Psychology, University of Stirling, Stirling, Scotland; 3 Department of Marketing, Innovation and Organisation, Ghent University, Ghent, Belgium; 4 Department of Psychology, Neuroscience & Behaviour, McMaster University, Hamilton, Canada

**Keywords:** Psychology, Human behaviour

## Abstract

There is growing concern that artificial intelligence conversational agents (e.g., Siri, Alexa) reinforce voice-based social stereotypes. Because little is known about social perceptions of conversational agents’ voices, we investigated (1) the dimensions that underpin perceptions of these synthetic voices and (2) the role that acoustic parameters play in these perceptions. Study 1 (N = 504) found that perceptions of synthetic voices are underpinned by Valence and Dominance components similar to those previously reported for natural human stimuli and that the Dominance component was strongly and negatively related to voice pitch. Study 2 (N = 160) found that experimentally manipulating pitch in synthetic voices directly influenced dominance-related, but not valence-related, perceptions. Collectively, these results suggest that greater consideration of the role that voice pitch plays in dominance-related perceptions when designing conversational agents may be an effective method for controlling stereotypic perceptions of their voices and the downstream consequences of those perceptions.

## Introduction

Perceptions of human faces and voices influence important social outcomes^1,2^. For example, people prefer to date, mate with, hire, and vote for individuals perceived as being particularly attractive^1,2^. These social perceptions (e.g., our impressions of other people’s attractiveness or trustworthiness) can also influence life-or-death outcomes. For example, untrustworthy-looking defendants are more likely to receive death sentences^3^.

Several recent studies suggest that social perceptions are underpinned by two key dimensions. For example, Principal Component Analysis (PCA) of ratings of natural human faces on a variety of different traits on which people spontaneously assess faces (trustworthiness, emotional stability, responsibility, sociability, caringness, attractiveness, intelligence, confidence, weirdness, unhappiness, meanness, aggressiveness, dominance) reveals two key underlying dimensions^4,5^. The first of these dimensions, often labelled valence, is highly correlated with ratings of pro-social traits, such as trustworthiness, and is thought to reflect perceptions of an individual’s willingness to inflict harm on others^4,5^. The second of these dimensions, often labelled dominance, is highly correlated with traits such as dominance and aggressiveness and is thought to reflect perceptions of an individual’s capacity to inflict harm on others^4,5^. Comparable dimensions have also been observed in work that used similar data-reduction methods to reveal the dimensions that underpin social perceptions of natural human voices^6,7^.

Conversational agents (e.g., Siri and Alexa) have become ubiquitous and communicate with users via voices generated by artificial intelligence algorithms (i.e., synthetic voices). A recent report by UNESCO (United Nations Educational, Scientific and Cultural Organization) proposed that these synthetic voices have the potential to reinforce voice-based gender stereotypes and argued that research is urgently needed to ameliorate this issue^8^. Crucially, understanding how people perceive synthetic voices is necessary to both understand and address this issue. While there is a large literature examining the factors that influence perceptions of natural social stimuli (natural human faces and voices), it is unclear whether our understanding of the factors that shape perceptions of natural social stimuli is applicable to perceptions of the synthetic voices employed by conversational agents. Indeed, previous work has shown qualitative differences in how people process and perceive natural human faces and synthetic faces^9,10^. Such results suggest that the factors that underpin perceptions of natural human stimuli may not necessarily underpin perceptions of synthetic stimuli. Indeed, some previous research suggests that natural human voices are perceived to be more expressive, understandable, and likeable than synthetic voices, suggesting that people can distinguish between natural human voices and synthetic voices and may process them differently^11^. However, other work suggests that the expectation that synthetic voices possess robotic qualities can also cause listeners to have difficulty when they are asked to classify voices as natural or artificial^12^. In light of these points, Study 1 first investigated whether trait-ratings of the synthetic voices used by conversational agents are underpinned by valence and dominance dimensions similar to those observed for natural human stimuli in previous work^4,5^.

Work on social perceptions of natural human voices has suggested that acoustic properties of voices can be key predictors and determinants of voice perceptions^13,14^. The majority of this work has focused on fundamental frequency (the acoustic correlate of perceived pitch) and formant frequencies (a correlate of vocal tract length and body size,^13^). For example, studies have reported strong negative relationships between dominance perceptions and voice pitch and/or that voices in which pitch was lowered are perceived to be more dominant than voices in which pitch was raised^13,14^. In light of findings such as these, Study 1 also investigated possible relationships between the dimensions that underpin social perceptions of synthetic voices and both pitch and formant frequencies.

Study 2 built directly on our results of Study 1 by testing whether Study 1’s results for correlations between social perceptions and measured acoustic properties of synthetic voices also occur when we experimentally manipulated acoustic parameters of voices. Whereas stimuli in Study 1 were individual words, stimuli in Study 2 were full sentences.

Collectively, these studies may be an important first step in identifying how appropriate the large literature on perceptions of natural social stimuli is to understand how we perceive and interact with the type of synthetic voices employed by artificial conversational agents. They may also identify mechanisms through which designers of conversational agents can better control stereotypic perceptions of conversational agents’ voices and the downstream consequences of these perceptions.

## Study 1

Study 1 aimed to (1) identify the perceptual dimensions that underpin social judgments of synthetic voices and (2) investigate how these perceptual dimensions are related to voice pitch and formant frequencies.

## Methods

### Ethics

All procedures were approved by the School of Psychological Sciences and Health (University of Strathclyde) Ethics Commitee, all work was undertaken in accordance with the Declaration of Helsinki, and all participants provided informed consent.

### Stimuli

Forty-six synthetic (i.e., computer-generated) voices (17 male, 29 female) were downloaded from online voice synthesis platforms (28 voices) or were provided to us by companies working on synthetic voice production (18 voices). Because studies that measured pitch and formant frequencies from recordings of natural human voices have generally used /α/, /ε/, /i/, /o/, and /u/ vowel sounds^13^, the voices used in the current study spoke the words *‘*Father, See, Bet, Note, Boot*’*. All stimuli were amplitude normalized to 70 dB prior to both acoustic analyses and collection of trait ratings. Voices spoke in English and reflected a range of accents. Stimuli are publicly available at https://osf.io/4zgrf/.

### Trait-rating procedure

Five hundred and six participants (213 men, 291 women, two participants did not report their gender; mean age = 34.0 years, standard deviation = 11.0 years) were recruited through the Prolific participant recruitment platform. All participants reported having English as their first language.

Participants were randomly allocated to rate all 46 voice stimuli for one of 17 traits (trustworthy, emotionally stable, responsible, sociable, caring, attractive, intelligent, confident, weird, unhappy, mean, aggressive, dominant, competent, old, masculine, feminine) on a 1 (not at all) to 7 (very) scale. The order in which stimuli were presented for rating was fully randomized and participants could play each voice as many times as they wanted to before rating it. Participants had to click a play button to play each voice and could not rate the voice and proceed to the next trial until the voice recording had been played in full. Ratings were made by clicking on labelled buttons, preventing participants from entering invalid responses. Mean stimulus duration was 3.42 s (SD = 0.42 s). Traits were chosen to reflect those used in previous studies of the dimensions underpinning perceptions of social stimuli (trustworthy, emotionally stable, responsible, sociable, caring, attractive, intelligent, confident, weird, unhappy, mean, aggressive, dominant, old,^4,5,7^). Masculinity and femininity were also included as separate traits, rather than being treated as opposite ends of a single continuum, because of recent work suggesting that they can have independent effects on perceptions of social stimuli and that including these characteristics as separate traits increased the predictive accuracy of models of social perception^15^. Competence was included because of research suggesting it plays an important role in a range of social outcomes (see, e.g.,^16^ for a recent review of this literature). Age, masculinity, and femininity were included because they had been included in previous research that used similar data-reduction methods to identify the perceptual dimensions underpinning social judgments^7,17^. Words spoken by each voice were presented in a single file for rating.

Table 1 shows the number of raters who rated stimuli on each trait, inter-rater agreement for those ratings (indicated by Cronbach’s alpha), the mean rating for each trait, and standard deviation. Because inter-rater agreement in ratings was relatively high for all traits, we calculated the mean rating for each voice. These mean ratings were calculated separately for each trait and were used in subsequent analyses. All data and analysis code are publicly available at https://osf.io/4zgrf/.

**Table 1:** Cronbach’s alpha for each trait, number of raters who rated voices for each trait, mean rating for each trait, and standard deviation (SD) in Study 1.

Trait	Cronbach’s alpha	Number of raters	Mean	SD
Aggressiveness	0.915	29	2.62	1.46
Attractiveness	0.890	30	3.55	1.73
Caringness	0.889	30	3.54	1.70
Competence	0.926	30	4.37	1.60
Confidence	0.913	29	4.19	1.70
Dominance	0.951	30	3.87	1.68
Emotional stability	0.901	30	4.25	1.70
Femininity	0.991	30	4.07	2.13
Intelligence	0.913	29	4.07	1.65
Masculinity	0.996	29	3.20	2.25
Meanness	0.808	30	3.19	1.74
Old	0.963	30	3.94	1.47
Responsibility	0.919	30	4.01	1.71
Sociable	0.859	30	3.74	1.67
Trustworthiness	0.869	30	3.87	1.70
Unhappiness	0.767	30	3.49	1.64
Weirdness	0.923	30	3.93	1.85

### Acoustic analyses

Vowel sounds were extracted from each voice recording and analyzed using PRAAT^18^. The methods used to measure pitch (f0) and formant frequencies were identical to those used to measure acoustic properties of natural speech in recent work on social judgments of human voices^13^. f0, f1, f2, f3, and f4 were measured separately from each vowel sound. Next, all measures were converted to z-scores. Standardized f0, averaged across vowel sounds, was then used as our measure of pitch in our statistical analyses. The remaining standardized measures (f1, f2, f3, and f4) were first averaged for each vowel sound and then averaged across vowel sounds. This latter score was used as our measure of formants in our statistical analyses.

## Results

All analyses were carried out using R^19^ and the packages tidyverse 1.3.0^20^, readxl 1.3.1^21^, psych 2.0.12^22^, paran 1.5.2^23^, kableExtra 1.3.4^24^, knitr 1.31^25^, and jtools 2.1.3^26^. All data and analysis code are publicly available at https://osf.io/4zgrf/.

First, mean trait ratings for each voice were subject to Principal Component Analysis (PCA) with oblimin rotation. This analysis revealed two Principal Components (PCs), explaining 45 and 29% of the variance in ratings, respectively. Factor loadings of the individual traits on both PCs are shown in Table 2.

**Table 2:** Correlations between each trait and scores on the Valence and Dominance components in Study 1.

Trait	Valence PC	Dominance PC
Aggressiveness	− 0.135	**0.866**
Attractiveness	**0.897**	− 0.162
Caringness	**0.644**	**− 0.587**
Competence	**0.937**	0.185
Confidence	**0.797**	0.297
Dominance	0.388	**0.867**
Emotional stability	**0.888**	0.094
Femininity	0.094	**− 0.834**
Intelligence	**0.845**	0.232
Masculinity	0.005	**0.850**
Meanness	0.026	**0.792**
Old	0.139	**0.721**
Responsibility	**0.884**	0.264
Sociable	**0.845**	− 0.350
Trustworthiness	**0.939**	− 0.179
Unhappiness	− 0.462	0.311
Weirdness	**− 0.828**	0.061

Correlations with absolute values larger than 0.5 are bolded.

Consistent with previous research using natural human voices and faces as stimuli^4,5,7^, the first PC was highly correlated with pro-social traits, such as trustworthiness, competence, responsibility, emotional stability, and sociableness, but weakly correlated with dominance and aggressiveness, and the second PC was highly correlated with dominance and aggressiveness, but weakly correlated with trustworthiness, competence, responsibility, emotional stability, and sociableness. Following previous research showing this pattern of results, we labelled these PCs Valence and Dominance, respectively. There was a non-significant, very weak, positive correlation between scores on both these components (*r*(46) = 0.07, *p* = 0.655).

Next, we used regression analyses to investigate possible relationships between PC scores and the acoustic characteristics pitch and formants. Separate models were run for Valence and Dominance PC scores. PC scores were our outcome variable. Predictors were voice gender (effect coded so that − 0.5 = male and 0.5 = female), pitch, formants, all two-way interactions, and the three-way interaction.

**Figure 1:**
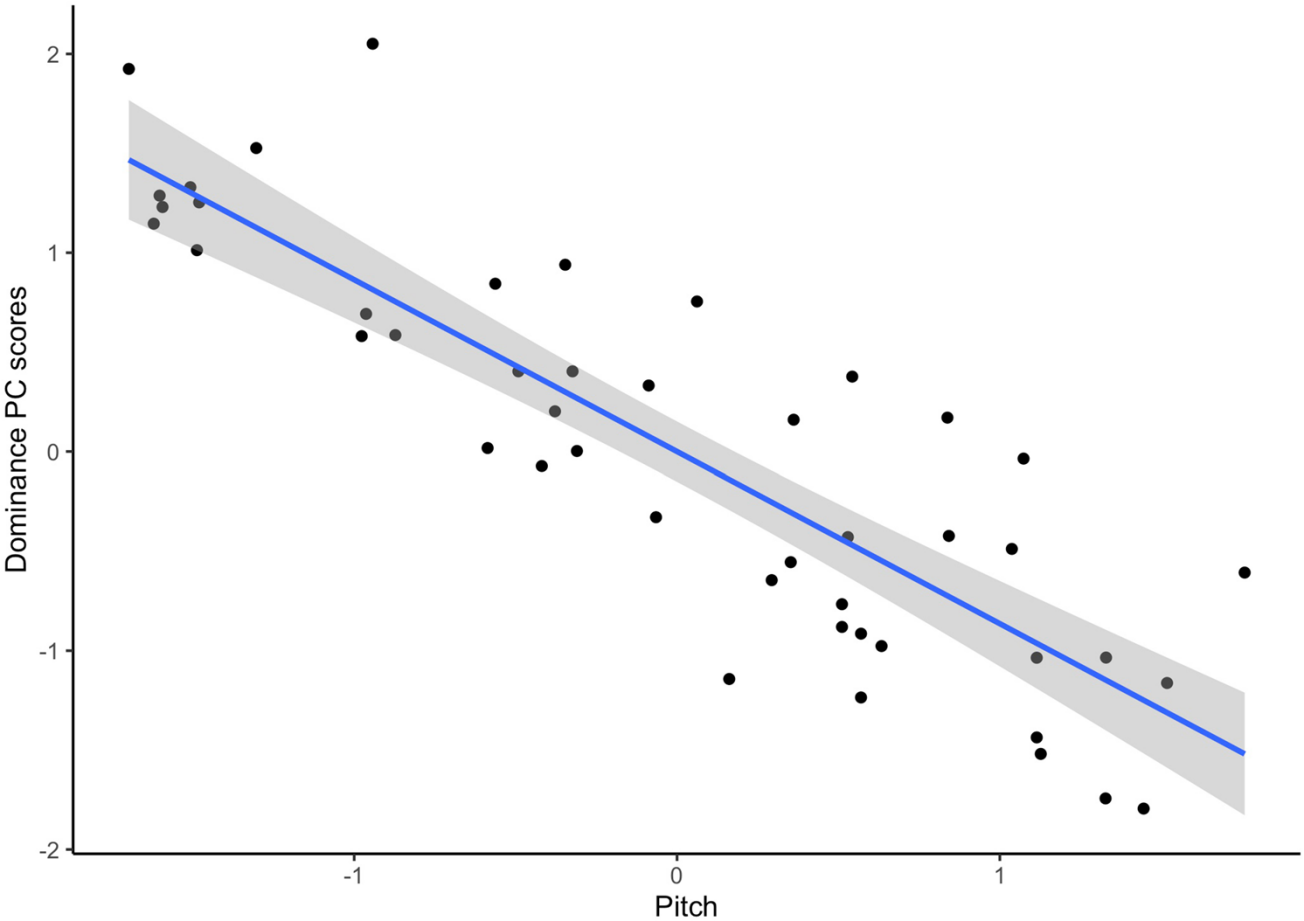
The significant negative relationship between pitch and Dominance PC scores in Study 1. Axes show standardized scores.

Full results from these regression analyses are summarized in Table 3 (Valence PC scores) and Table 4 (Dominance PC scores). For Valence, the regression model was not significant (*F*(7,38) = 1.04, adjusted R squared = 0.01, *p* = 0.42) and the model showed no significant effects for any of the predictors (Table 3). For Dominance, the regression model was significant (F(7, 38) = 21.8, adjusted R squared = 0.76, *p* < 0.001) and a strong significant negative effect of voice pitch was observed (Table 4). Figure 1 shows the negative relationship between pitch and Dominance PC scores. Pitch explained 74% of the variance in Dominance PC scores.

**Table 3:** Results of regression analysis of Valence PC scores in Study 1.

	Unstandardized estimate	Standard error	t	*p*
Intercept	0.46	0.45	1.02	0.315
Pitch	− 0.36	0.35	− 1.01	0.317
Formants	1.04	0.73	1.43	0.162
Voice sex	− 0.82	0.91	− 0.91	0.370
Pitch × formants	0.45	0.53	0.86	0.397
Pitch × voice sex	− 0.35	0.71	− 0.50	0.621
Formants × voice sex	− 1.54	1.45	− 1.06	0.297
Pitch × formants × voice sex	− 0.30	1.06	− 0.29	0.776

**Table 4:** Results of regression analysis of Dominance PC scores in Study 1.

	Unstandardized estimate	Standard error	t	*p*
Intercept	− 0.13	0.22	− 0.60	0.550
Pitch	− 0.76	0.17	− 4.40	< 0.001
Formants	− 0.50	0.35	− 1.42	0.165
Voice sex	0.13	0.44	0.29	0.770
Pitch × formants	− 0.26	0.26	− 1.01	0.321
Pitch × voice sex	0.26	0.35	0.74	0.461
Formants × voice sex	0.57	0.71	0.80	0.428
Pitch × formants × voice sex	0.23	0.52	0.45	0.658

Robustness checks in which ratings of male and female voices were analyzed separately showed the same patterns of results as the analyses described above. That is, separate PCAs of ratings of male and female voices revealed Valence and Dominance PCs that were similar to those we obtained from PCA of all voices. Further analyses also showed that, for both male and female voices, pitch was strongly and negatively related to scores on the Dominance PC. Full results for these analyses are given in the supplemental analyses for Study 1 (https://osf.io/4zgrf/).

## Study 2

Study 1 demonstrated that trait-ratings of synthetic voices are underpinned by Valence and Dominance components similar to those previously reported for perceptions of natural human stimuli. Study 1 also found a strong negative relationship between pitch and scores on the Dominance PC. By contrast, there was no significant relationship between pitch and scores on the Valence PC. Study 2 attempted to validate the results of Study 1 by investigating the effects of manipulating voice pitch on perceptions of dominance and aggressiveness (i.e., the two individual traits most strongly correlated with Dominance PC scores in Study 1) and perceptions of trustworthiness and competence (i.e., the two individual traits most strongly correlated with Valence PC scores in Study 1). Whereas voices in Study 1 spoke individual words, stimuli in Study 2 spoke a full sentence commonly used in studies of social judgments of natural voices that have used full sentences as stimuli (“When the sunlight strikes raindrops in the air, they act as a prism and form a rainbow.”).

## Methods

### Stimuli

Stimuli were manufactured from recordings of the same 46 voices used in Study 1, this time speaking the sentence “When the sunlight strikes raindrops in the air, they act as a prism and form a rainbow”. This sentence was chosen because it has been used in previous studies of social judgments of natural voices^27,28^.

Two versions of each recording were created; one version (raised-pitch version) in which the pitch of the recording was raised by 0.5 equivalent rectangular bandwidths (ERBs) and another version (lowered-pitch version) in which the pitch of the recording had been lowered by 0.5 ERBs. The ERB scale corrects for the difference between perceived pitch and actual fundamental frequency. Mean pitch for the lowered-pitch versions was 142.06 Hz (*SD* = 8.71 Hz) and mean pitch for the raised-pitch versions was 184.83 Hz (*SD* = 9.09 Hz). Pitch was manipulated using the same methods used in previous studies of the effect of manipulating pitch on social judgments of natural voices^29–31^. Only the raised- and lowered-pitch versions were used in the study. All stimuli were amplitude normalized to 70 dB prior to collection of trait ratings. Stimuli are publicly available at https://osf.io/4zgrf/. Voices spoke in English and reflected a range of accents.

### Trait-rating procedure

One hundred and sixty participants (59 men, 93 women, eight participants did not report their gender; mean age = 31.5 years, standard deviation = 11.6 years) were recruited through the Prolific participant recruitment platform. All participants reported having English as their first language.

Participants were randomly allocated to rate all 92 voice stimuli (the 46 raised-pitch and 46 lowered-pitch versions) for either competence (36 raters), trustworthiness (40 raters), dominance (44 raters), or aggressiveness (40 raters) on a 1 (not at all) to 7 (very) scale. The order in which stimuli were presented for rating was fully randomized and participants could play each voice as many times as they wanted to before rating it. Participants had to click a play button to play each voice and could not rate the voice and proceed to the next trial until the voice recording had been played in full. Ratings were made by clicking on labelled buttons, preventing participants from entering invalid responses. Mean stimulus duration was 5.40 s (SD = 0.42 s). Inter-rater agreement was high for all traits (all Cronbach’s alpha > 0.93).

## Results

All analyses were carried out using R^19^ and the packages tidyverse 1.3.0^20^, broom.mixed 0.2.6^32^, lmerTest 3.1-3^33^, psych 2.0.12^22^, and jtools 2.1.3^26^. All data and analysis code are publicly available at https://osf.io/4zgrf/.

We used linear mixed models to investigate possible effects of the pitch manipulation on competence, trustworthiness, dominance, and aggressiveness ratings. Separate models were run for each trait. Ratings were the outcome variables and predictors were voice gender (effect coded so that − 0.5 = male and 0.5 = female), pitch manipulation (effect coded so that lowered pitch = − 0.5 and raised pitch = 0.5), and the interaction between voice gender and pitch manipulation. The models included random intercepts for participant and stimulus. Random slopes were specified maximally.

**Figure 2:**
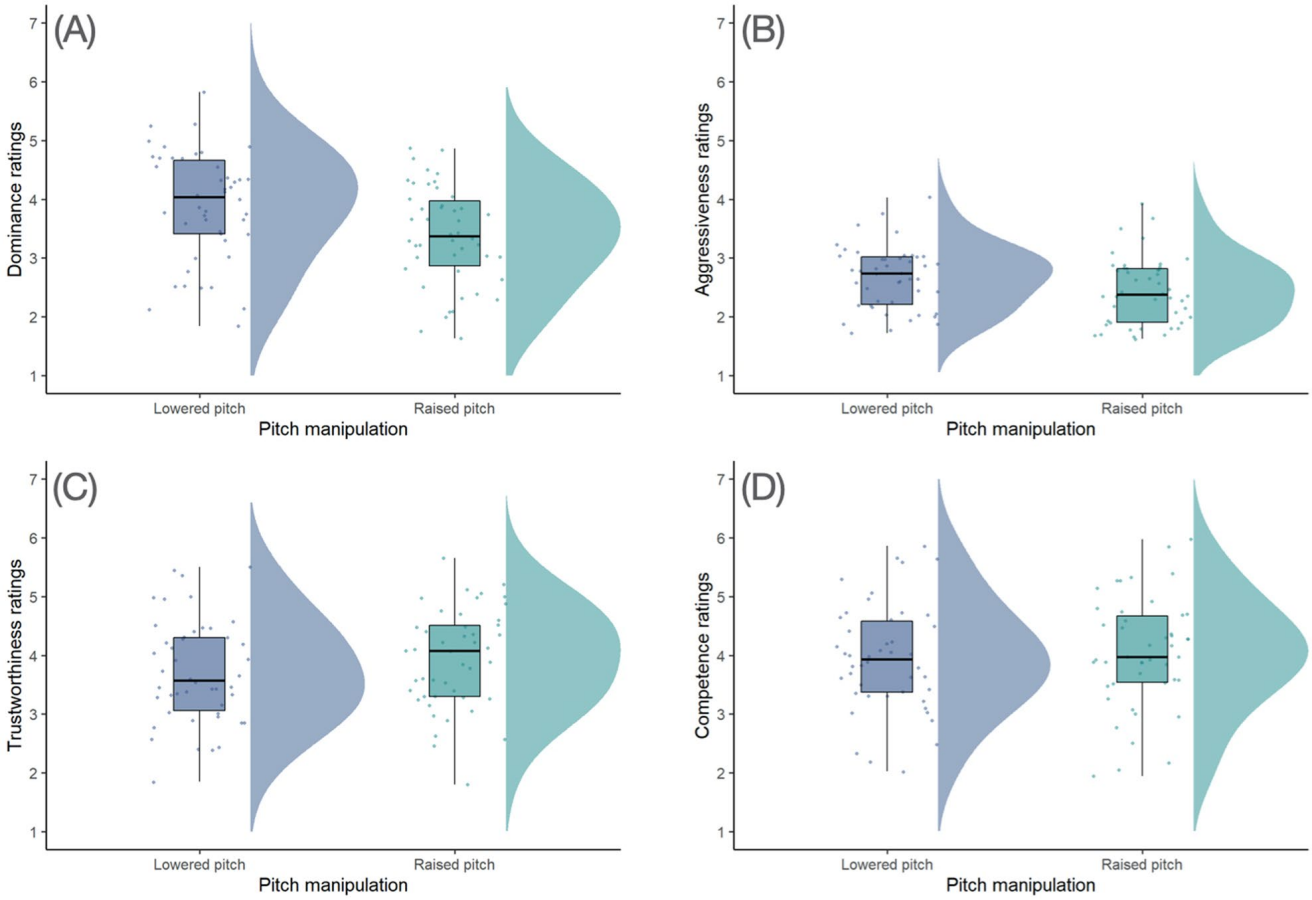
The effect of pitch manipulation on (**A**) dominance, (**B**) aggressiveness, (**C**) trustworthiness, and (**D**) competence ratings in Study 2. Significant effects of pitch were observed for dominance and aggressiveness ratings, but not trustworthiness or competence ratings. The points and distributions represent the average rating for each voice. The box plots show the median, first and third quartile, and the minimum and maximum dominance rating for low (purple) and high (green) pitch.

Results of these analyses are summarized in Table 5 (competence ratings), Table 6 (trustworthiness ratings), Table 7 (dominance ratings), and Table 8 (aggressiveness ratings). While our analyses of competence and trustworthiness ratings showed no significant effect of pitch manipulation, our analyses of dominance and aggressiveness ratings showed that the lowered-pitch versions of voices were judged significantly more dominant and aggressive than the raised-pitch versions. Figure 2 shows the negative effects of the pitch manipulation on dominance and aggressiveness perceptions.

**Table 5:** Results of our analysis of competence ratings in Study 2.

	Unstandardised estimate	Standard error	df	t	*p*
Intercept	4.04	0.16	75.4	25.40	< 0.001
Pitch	0.07	0.20	94.3	0.34	0.732
Voice gender	− 0.38	0.20	98.4	− 1.88	0.063
Pitch × voice gender	0.19	0.39	91.6	0.50	0.619

**Table 6:** Results of our analysis of trustworthiness ratings in Study 2.

	Unstandardised estimate	Standard error	df	t	*p*
Intercept	3.85	0.12	113.0	32.10	< 0.001
Pitch	0.20	0.19	97.1	1.05	0.298
Voice gender	− 0.15	0.19	97.8	− 0.82	0.413
Pitch × voice gender	0.19	0.37	91.8	0.51	0.610

**Table 7:** Results of our analysis of dominance ratings in Study 2.

	Unstandardised estimate	Standard error	df	t	*p*
Intercept	3.77	0.13	87.3	28.40	< 0.001
Pitch	− 0.53	0.17	103.0	− 3.20	0.002
Voice gender	− 1.00	0.19	123.0	− 5.31	< 0.001
Pitch × voice gender	0.14	0.32	91.5	0.45	0.651

**Table 8:** Results of our analysis of aggressiveness ratings in Study 2.

	Unstandardised estimate	Standard error	df	t	*p*
Intercept	2.63	0.17	47.4	15.70	< 0.001
Pitch	− 0.22	0.10	94.4	− 2.12	0.037
Voice gender	− 0.57	0.13	111.0	− 4.55	< 0.001
Pitch × voice gender	− 0.07	0.20	91.0	− 0.35	0.724

## Discussion

PCA of ratings of synthetic voices used by conversational agents on a range of traits (trustworthiness, emotional stability, responsibility, sociability, caringness, attractiveness, intelligence, confidence, weirdness, unhappiness, meanness, aggressiveness, dominance, competence, age, masculinity, femininity) produced two components. The first component, which explained the bulk of the variance in ratings, was highly correlated with ratings of pro-social traits, such as trustworthiness, competence, responsibility, emotional stability, and sociable, and weakly correlated with dominance and aggressiveness ratings. The second component, which explained substantially less of the variance in ratings, was highly correlated with dominance and aggressiveness ratings and weakly correlated with trustworthiness, competence, responsibility, emotional stability, and sociable ratings. This pattern of results is extremely similar to those obtained when ratings of natural human faces and voices were subject to PCA in previous studies^4–7^, suggesting that social perceptions of synthetic voices are underpinned by valence and dominance dimensions similar to those previously found to underpin social perceptions of natural human stimuli.

Analyses of acoustic properties of synthetic voices and their relationship to the PCs in Study 1 revealed a strong negative correlation between pitch and scores on the Dominance component. Consistent with results of this correlational analysis, experimentally manipulating the pitch of synthetic voices in Study 2 had a strong effect on dominance and aggressiveness perceptions (the two traits most strongly correlated with the Dominance component). Synthetic voices with lowered pitch were judged significantly more dominant and aggressive than those with raised pitch (Fig. 2). These results are consistent with previously reported results for both measured and manipulated pitch and dominance perceptions of natural human voices^13,14^.

Previous studies of formant frequencies and social perceptions of natural human voices have typically reported that voices with lower formant frequencies are perceived to be more dominant^34^. Previous studies of perceptions of natural human voices have also often reported associations between attractiveness and both voice pitch and formant frequencies^31,34^. In our study, we did not observe significant associations between scores on the Dominance PC and measured formants (Study 1) or between scores on the Valence PC (very highly correlated with attractiveness) and measured pitch or formants (Study 1). It is currently unclear whether these potential differences in results for synthetic voices in the current studies and natural human voices in previous studies reflect differences in how these two classes of stimuli are perceived or methodological differences among studies (e.g., larger number of voices tested in studies of natural human voices). While further work is needed to address this issue, our results clearly indicate that pitch is particularly strongly related to (and, as our results from Study 2 indicate, directly influences) dominance-related perceptions of synthetic voices. Indeed, in Study 1, pitch explained ~ 74% of the variance in Dominance PC scores. In a recent study of natural human voices, Schild et al.^35^ found that trustworthiness ratings were not significantly correlated with pitch, but that pitch was a very good predictor of dominance ratings. Our findings for conversational agents’ voices very closely align with Schild et al.’s results.

Three key aspects of the research reported here suggest that our results are likely to have good generalisability. First, we show that dominance-related perceptions of voices are both highly correlated with voice pitch (Study 1) and directly influenced by experimentally manipulating voice pitch (Study 2). This pattern of results suggests that our results generalise well across two different types of study design. Second, in Study 2 we analysed responses using linear mixed models that take into account variability in responses across both raters and stimuli. This analytical strategy is known to produce results that generalise better to new sets of stimuli than those of analytical approaches in which responses are aggregated across stimuli^36^. Third, the similarity in our results for dominance-related perceptions of synthetic voices to those reported elsewhere for natural human voices suggest the large literature on dominance-related perceptions of natural human voices is likely to be useful for understanding perceptions of synthetic voices.

To summarize, our results for social perceptions of conversational agents’ synthetic voices highlight two clear similarities in the characteristics of judgments of synthetic voices and those previously reported for natural human stimuli. Like natural human voices, our results show that perceptions of synthetic voices appear to be underpinned by Valence and Dominance dimensions and that dominance-related perceptions are both strongly related to voice pitch and directly affected by experimental manipulations of voice pitch. Collectively, these results suggest that greater consideration of the role that voice pitch plays in dominance-related social perceptions when designing conversational agents will be effective in controlling stereotypic perceptions of their voices and the downstream consequences of those perceptions.

## Data Availability

Data, analysis code, and stimuli for both studies are publicly available on the Open Science Framework (https://osf.io/4zgrf/).
